# Transcriptome wide analysis of long non‐coding RNA‐associated ceRNA regulatory circuits in psoriasis

**DOI:** 10.1111/jcmm.16703

**Published:** 2021-06-02

**Authors:** Jingxia Lin, Xuefei Li, Fangfei Zhang, Lei Zhu, Yongfeng Chen

**Affiliations:** ^1^ Dermatology Hospital Southern Medical University Guangzhou Guangdong China

**Keywords:** ceRNA, IFN gamma signalling pathway, JAK/STAT pathway, long non‐coding RNAs, RNA‐seq

## Abstract

Long non‐coding RNAs (lncRNAs) play critical roles in regulating immune‐associated diseases and chronic inflammatory disorders. Here, we found that lncRNAs involve in the pathogenesis of psoriasis through integrative analysis of RNA‐seq data sets from a psoriasis cohort. Then, lncRNA‐protein‐coding genes (PCGs) co‐expression network analysis demonstrated that lncRNAs extensively interact with IFN‐γ signalling pathway‐associated genes. Further, we validated 3 lncRNAs associate with IFN‐γ signalling pathway activation upon IFN‐γ stimulated in HaCaT cells, and loss of function experiments indicate their functional roles in the activation of inflammatory cytokine genes. Additionally, microRNA target screening analysis showed that lncRNAs may regulate JAK/STAT pathway activity through complete endogenous RNA (ceRNA) mechanism. Further experimental validation of PRKCQ‐AS1/STAT1/miR‐545‐5p regulatory circuitry showed that lncRNAs regulate the expression of JAK/STAT signalling pathway genes through competing for miR‐545‐5p. In summary, our results demonstrated that dysregulation of lncRNA‐JAK/STAT pathway axis promotes the inflammation level in psoriasis and thus provide potential therapeutic targets for psoriasis treatments.

## 1 INTRODUCTION

Long non‐coding RNA (lncRNA) are a diverse class of transcripts with a length >200 nucleotides that do not encode a protein, and it has gained widespread attention in recent years as a potentially new and crucial layer in various physiological processes.^1^ Psoriasis is a common chronic autoimmune disease affecting 0.51%‐11.43% of adults. High immune infiltration and hyperproliferation of keratinocytes are significant characteristics of psoriasis.^2^ Psoriasis can be conceptualized as an exaggerated physiological response to epithelial damage, and keratinocytes play a critical role in chronic inflammatory processes.^3^ Recent reports have shown that lncRNAs play an essential role in the immune‐associated disorders such as psoriasis and atopic dermatitis (AD).^4^, ^5^ Numerous studies have demonstrated that lncRNAs are associated with the differentiation and activation of immune cells, including dendritic cells, neutrophils, T cells and macrophages.^6^ Elevation of lncRNAs was observed upon cytokine treatment in cultured keratinocytes.^7^ In addition, Li et al demonstrated that lncRNA H19 plays an essential role in keratinocyte differentiation through interacting with miR‐130b‐3p.^8^ Although many researchers made great efforts to dissect the functional mechanism of lncRNA in psoriasis, only a few of them have been experimental validated so far.^9^ Therefore, the functional roles of lncRNAs in psoriasis require further investigations.

Interferons (IFNs) play an important role in innate immune responses through IFN‐γ signalling and activation of intracellular JAK/STAT signalling pathway.^10^ Disruption of the JAK/STAT signalling pathway might lead to various autoimmune diseases and inflammatory diseases.^11^, ^12^ Recent studies showed that the JAK/STAT pathway might involve inflammatory and neoplastic skin diseases, like psoriasis, atopic dermatitis, vitiligo and melanoma.^11^ Besides, the JAK/STAT signalling pathway is essential for the activation of a wide range of cytokines and growth factors, leading to critical cellular events, such as cell proliferation, differentiation and apoptosis.^13^ Psoriasis is a prototypic Th17 cell‐mediated autoimmune disease with high expression of IL‐17, TNF, IFN‐γ, IL‐22 and IL‐23.^14^ Numerous studies have shown that critical pathogenic mediators of psoriasis in the JAK/STAT signalling pathway.^8^, ^15^ For instance, Th1 cytokine IFN‐γ induces phosphorylation of STAT1 by Janus kinases (JAK).^13^ IFN‐γ induced the expression of numerous genes in the skin contributing to chronic inflammation and implicated in the pathogenesis of psoriasis.^16^ Additionally, STAT1 expression is elevated in lesion psoriatic skin, suggesting that the JAK/STAT signalling pathway is associated with psoriasis.^17^ Furthermore, IL‐23 produced by dendritic cells and macrophages promotes Th17 cell expansion and survival within psoriatic skin, engagement with its cognate receptors, resulting in activation of STAT3 and STAT4.^18^ However, whether lncRNAs participate in the activation of the JAK/STAT pathway in the onset of psoriasis remains mostly unknown.

In this study, we found that lncRNAs involve in the pathogenesis of psoriasis through integrative analysis of RNA‐seq data from a psoriasis cohort. Then, lncRNA‐protein‐coding genes (PCGs) co‐expression network analysis demonstrated that lncRNAs extensively interact with IFN‐γ signalling pathway‐associated genes. Further expression dynamic analysis and loss of function analysis demonstrated that 3 lncRNAs associate with the activation of JAK/STAT signalling pathway upon IFN‐γ stimulated in HaCaT cells, indicating their functional roles in promoting inflammatory cytokines production in psoriatic keratinocytes. Moreover, microRNA target screening analysis demonstrated that lncRNAs and JAK/STAT pathway‐associated genes co‐regulated by the same set of miRNAs, suggesting lncRNAs may regulate the JAK/STAT pathway through complete endogenous RNA (ceRNA) mechanism. Further experimental validation of PRKCQ‐AS1/STAT1/miR‐545‐5p regulatory circuitry showed that lncRNA might regulate the activity of the JAK/STAT signal pathway through the ceRNA mechanism. In conclusion, our results demonstrated the functional mechanism of lncRNAs in psoriasis, which will benefit our further understanding of the pathogenesis of various skin diseases.

## 2 MATERIALS AND METHODS

### 2.1 Data collection

RNA sequencing (RNA‐seq) and small RNA sequencing (small RNA‐seq) data sets derived from psoriasis and healthy cohort were downloaded from the NCBI Gene Expression Omnibus database (GEO)^19^ with accession number GSE54456, GSE63979 (RNA‐seq)^4^, ^20^ and GSE31037 (small RNA‐seq).^21^ Briefly, we obtained 189 RNA‐seq data sets from 99 psoriatic and 90 healthy punch biopsies, while 44 small RNA‐seq data sets from 24 psoriasis biopsies and 20 healthy controls. Raw sequencing reads were extracted from SRA files by SRA‐toolkits.^22^ All data sets used in this study were listed in Table S1.

### 2.2 RNA‐seq data analysis

We adopt a transcriptome analysis pipeline established in our previous studies.^23^, ^24^ Briefly, low‐quality sequencing reads were filtered using adapter Removal^25^ if read length <50 bps and quality value <3.^26^ The remaining reads were then aligned to the human reference genome (hg19) using STAR aligner.^27^ We then calculated gene expression levels of all of the transcripts as fragments per kilobase of exon per million fragments mapped (FPKM) by cufflinks.^28^ Differentially expressed genes were detected by comparing psoriasis and healthy group using DEseq2.^29^ Finally, genes were identified as differentially expressed genes using Benjamini‐Hochberg adjusted *P*‐value < .05 and | Log_2_ (Foldchange) | ≥ 2 as the cut‐off.

### 2.3 Small RNA‐seq data analysis

Small RNA‐seq from psoriasis and healthy groups was analysed using miRDeep2.^30^ Briefly, low‐quality reads were eliminated using Cutadapt (version 2.10)^31^; the filtered reads were then aligned to miRbase and the human reference genome (hg19). The expression level of each microRNA is quantified as Tag Per Millions reads (TPM). The differentially expressed small RNA and microRNAs were identified if | Log_2_ (Foldchange) | of TPM is higher than 2.0 and the adjusted *P*‐values are less than *P* < .05.^32^

### 2.4 Construction of the lncRNA‐PCG co‐expression network

A gene expression matrix was constructed by integrating gene expression profiles from 189 RNA‐seq data sets of 99 psoriatic and 90 healthy punch biopsies. We then applied WGCNA to construct gene co‐expression network using the established gene expression matrix as input.^33^ The weak co‐expression gene pairs were eliminated with adjacency threshold higher than 0.02 and interacting with at least one lncRNA molecule, resulting in a reliable lncRNA‐PCG co‐expression network.

### 2.5 Identification of ceRNA pairs

The differentially expressed lncRNAs, protein‐coding genes (PCGs) and miRNAs were used to establish ceRNA interacting pairs. Briefly, we first screen potential miRNA target binding sites at 3’ UTR of PCGs and the full length of lncRNAs using Miranda^34^ with default parameters to identify ceRNA pairs among lncRNAs and PCGs. Then, we incorporated lncRNA‐PCG co‐expression profile and public databases to establish ceRNA pairs among lncRNAs and PCGs as follows: (1) retrieve miRNA‐PCG and miRNA‐lncRNA pairs if they at least exist in TarBase (version 6.0),^35^ miRTarBase (version 6.1)^36^ or miRecords (version 4)^37^; (2) retain lncRNA and PCG co‐regulated with the same set of miRNAs; (3) retain lncRNA‐PCG ceRNA pairs if both the lncRNA and PCG expression are negatively correlated with the same miRNA; (4) the expression of lncRNA is positive correlated with PCGs. The eligible ceRNA pairs among lncRNA and PCGs were integrated to form the final ceRNA network using an in‐house Perl script. The ceRNA network was imported into Cytoscape (version 3.3.0)^38^ (http://www.cytoscape.org) for visualization and network analyses.

### 2.6 Functional enrichment analysis

The differentially expressed genes were subjected to DAVID functional annotation pipeline for gene function enrichment analysis.^39^ KEGG pathways^40^ were identified if the adjusted *P*‐value was <.05. The pre‐ranked gene list with Log2 (Foldchange) was analysed by GSEA package against MSigDB.^41^

### 2.7 Statistical analysis

Data were analysed using GraphPad Prism (version 8; GraphPad Software). Data were represented as mean ± SD. All tests were two sided, and *P* < .05 was considered statistically significant.

### 2.8 Cell cultures

The spontaneously immortalized human keratinocyte named HaCaT (BNCC, Cat. NO. 3405CA93) were cultured in DMEM containing 10% foetal bovine serum (FBS), 2 mM L‐glutamine, 100 U mL 1 penicillin and 100 μg of streptomycin. HaCaT were cultured in 0 or 10 ng/mL of IFN‐γ. At indicated time‐points following cytokine exposure, cells were collected for quantification of STAT1 and PRKCQ‐AS1 expression.

### 2.9 RNA extraction and RT‐qPCR

Total RNA from HaCaT cells was extracted using TRIzol RNA Isolation reagent (Thermo Fisher Cat. NO. 191005) according to the manufacturer's instructions. The expression of lncRNAs was quantified by Real‐time PCR Mixture Assays with SYBR Premix Ex Taq II (TaKaRa Cat. NO. RR820A) using GAPDH as a control. All primers used in this study were listed in Table S2.

## 3 RESULTS

### 3.1 Identification of differentially expressed lncRNAs in psoriasis

**FIGURE 1:**
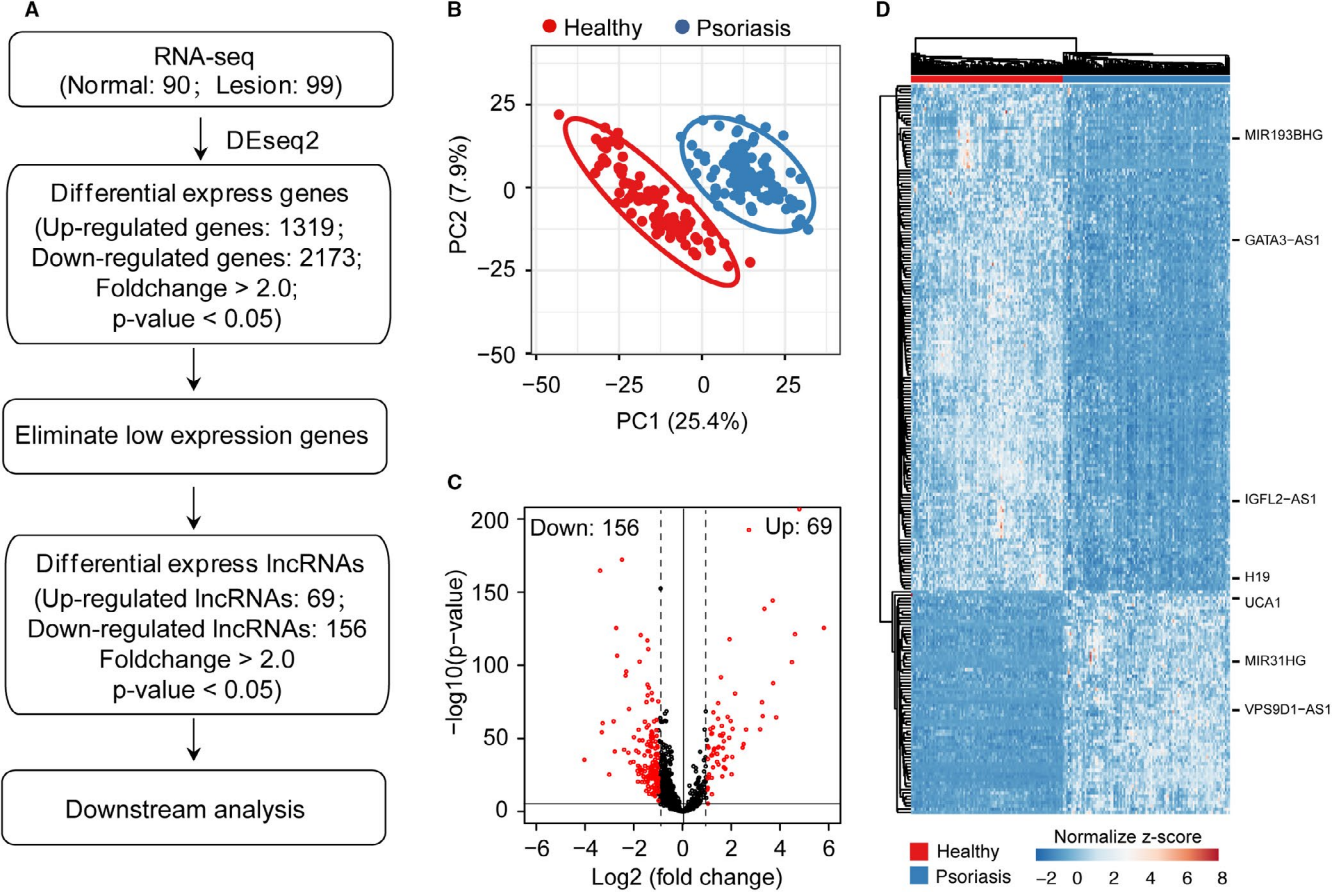
Identification of differentially expressed lncRNAs in psoriasis. A, Overview of RNA‐seq processing pipeline used in this study. B, Principal component analysis (PCA) showed that lncRNAs distinguished healthy skin (in red) from psoriatic skin (in blue). C, Scatterplot showed the differentially expressed lncRNAs in psoriasis compared with the healthy group. Differentially expressed lncRNAs are highlighted with red. D, Heatmap showed differentially expressed lncRNAs in psoriatic skin compared with healthy skin. Genes were filtered for moderate to high expression (raw signal > 500) and Fold Change > 2

Numerous studies have been shown that thousands of lncRNAs are differentially expressed in various disease conditions,^42^ but the biological function of most lncRNAs in skin diseases remains unknown. To gain more insights into the functional role of lncRNA in psoriasis, we analysed a set of RNA‐seq data derived from a psoriasis cohort including 90 normal skin and 99 psoriatic skin.^4^ Then, we applied a customized bioinformatics pipeline to identify differentially expressed genes (DEGs) and retrieve differentially expressed lncRNAs (DE‐lncRNAs) (Figure 1A). As a result, 1319 up‐regulated genes and 2173 down‐regulated genes were observed in psoriatic skin compared with normal skin (Table S3). Then, we carried out principal component analyses (PCA) among normal and psoriatic skin using all expressed lncRNAs in healthy and psoriatic skin. Expectedly, PCA analysis showed that the psoriasis group is clearly separated from the normal group, suggesting that the expression of lncRNA is significantly different between the healthy and psoriatic groups (Figure 1B). Further, we obtained DE‐lncRNAs including 69 up‐regulated and 156 down‐regulated‐lncRNAs by eliminating the low‐expressed lncRNAs (Figure 1C). Moreover, the heatmap analysis again confirms that DE‐lncRNAs successfully discriminate psoriatic group from healthy group (Figure 1D). Particularly, some lncRNAs have been experimentally validated in different diseases. For instance, lncRNA H19 and MIR31HG play an essential role in keratinocyte differentiation, indicating the potential function of H19 and MIR31HG in pathogenesis of psoriasis. Taken together, our RNA‐seq analysis demonstrated that dysregulation of lncRNA may participate in the inflammatory progression of psoriasis.

### 3.2 Long non‐coding RNAs may involve in IFN‐γ signaling pathway in psoriasis

**FIGURE 2:**
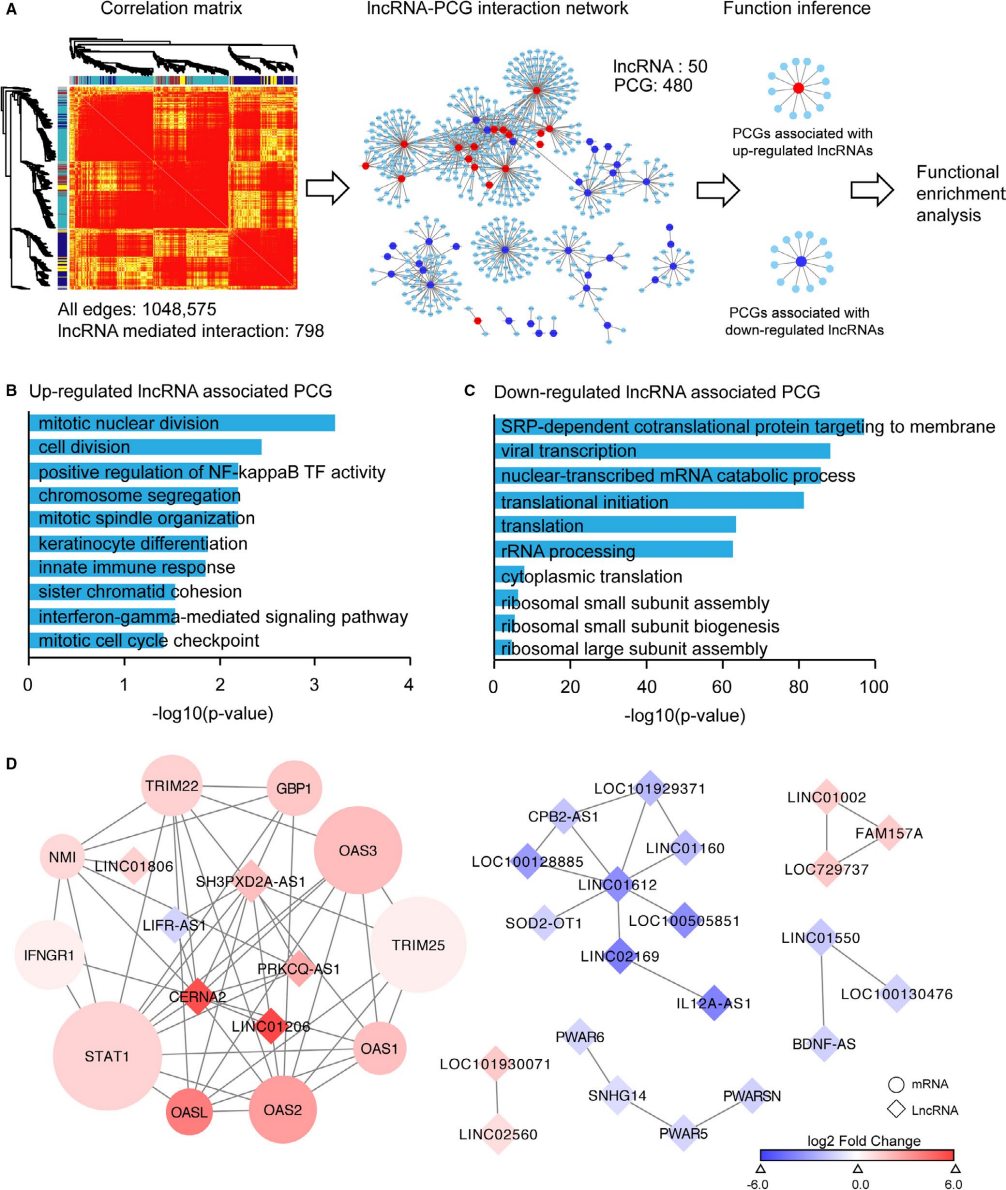
Long non‐coding RNAs may involve in IFN‐γ signalling pathway in psoriasis. A, Overview of lncRNA‐PCG interaction network analysis pipeline used in this study. Heatmap showed the hierarchical clustering dendrograms correspond to modules using RNA‐seq profiles in healthy and psoriasis group. The lncRNA‐PCG interaction network consists of 1,048,575 edges among 503 lncRNAs and 12,263 PCGs. The sub‐network consists of 798 edges among 50 lncRNAs and 480 mRNAs. B‐C, GO pathway enrichment analysis for PCGs associated with up‐regulated lncRNAs and down‐regulated lncRNAs. D, The mRNA‐lncRNA co‐expression network involved in IFN‐γ signalling pathway in psoriasis. The network consists of 62 edges among 27 lncRNA (square) and 10 mRNAs (circle)

Although extensive long non‐coding RNAs (lncRNAs) are elevated in psoriatic skin compared with normal skin, how those lncRNAs act their functional role still warrants further investigation.^42^ Towards this end, we sought to establish a lncRNA‐PCG interaction network including 503 lncRNAs and 12,263 PCGs in psoriasis group using WGCNA. Then, a subnet network with 50 DE‐lncRNAs and 480 PCGs was extracted (Table S4). Further, PCGs associated with up‐regulated lncRNAs and down‐regulated lncRNAs were subjected to gene functional enrichment analysis (Figure 2A). As a result, PCGs associated with up‐regulated lncRNAs were enriched in mitotic nuclear division, cell division, keratinocyte differentiation and IFN‐γ mediated signalling pathway etc, while PCGs associated with down‐regulated lncRNAs were enriched in SRP‐dependent co‐translational protein targeting to membranes, viral transcription and translational initiation etc (Figure 2B‐C). Intriguingly, PCGs associated up‐regulated lncRNA are enriched in innate immune response and IFN‐γ mediated signalling pathway, which are strongly associated with the pathological histology of psoriatic subjects (Figure 2B‐C; Table S5). When we retrieved the sub‐network among DE‐lncRNAs and PCGs involved in IFN‐γ signalling pathway in psoriasis, we found an extensive crosstalk among lncRNAs and the IFN‐γ signalling pathway (Figure 2D). Particularly, PRKCQ‐AS1, SH3PXD2A‐AS1 and CERNA2 are positively correlated to STAT1, indicating that the up‐regulated lncRNAs may participate in the progression of psoriasis through fine‐tuning the regulation of IFN‐γ signalling pathway.

### 3.3 Elevated lncRNA expression in keratinocytes upon IFN‐γ stimulation

**FIGURE 3:**
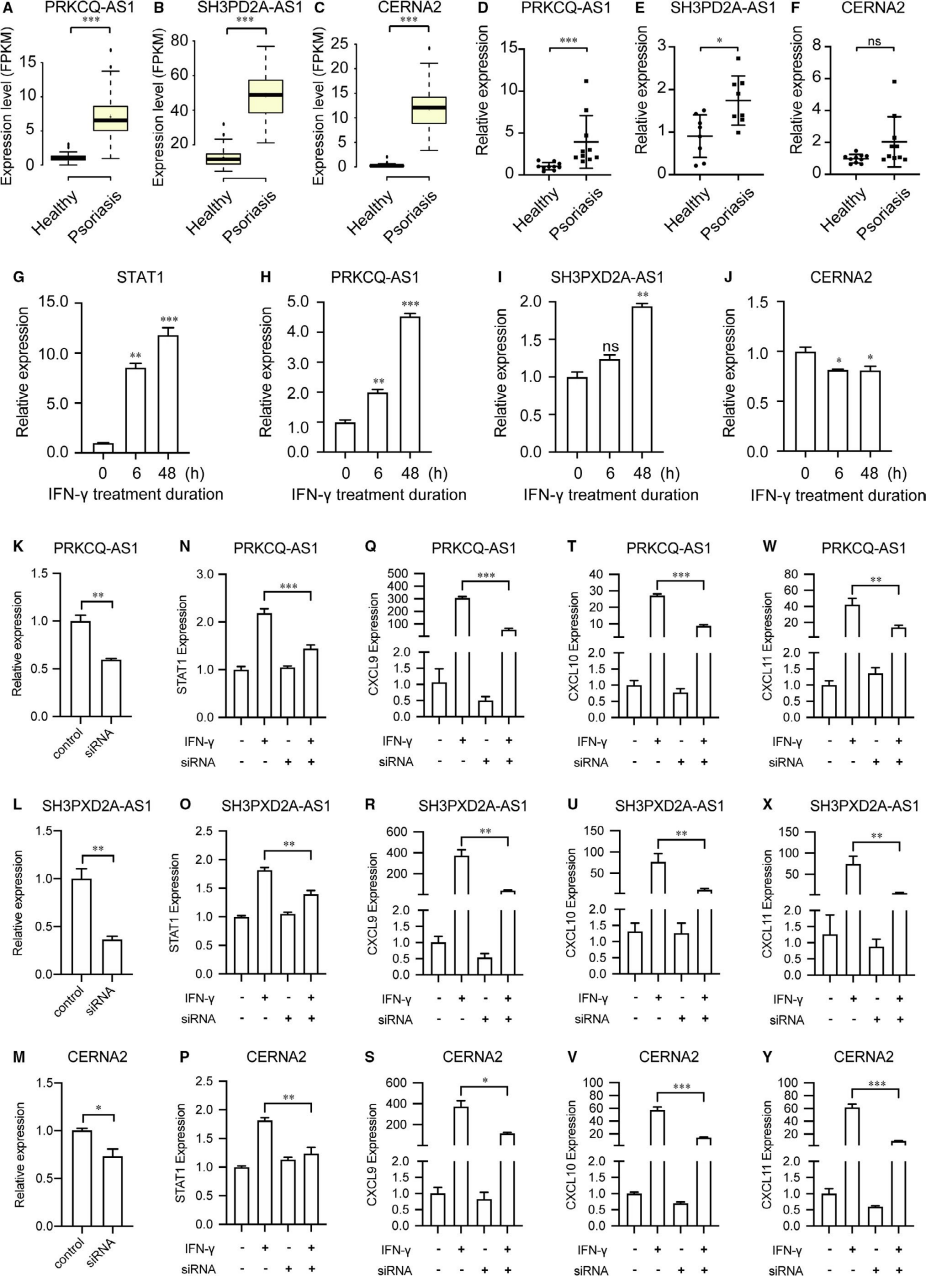
Elevated lncRNA expression in keratinocytes upon IFN‐γ stimulation. A‐C, 3 lncRNAs co‐expressed with STAT1 are significantly differentially expressed in psoriasis including PRKCQ‐AS1 (A), SH3PXD2A‐AS1 (B) and CERNA2 (C). D‐F, The expression of PRKCQ‐AS1 (D), SH3PXD2A‐AS1 (E) and CERNA2 (F), was determined by real‐time PCR (RT‐PCR) in clinical samples. G‐J, HaCaT cells were treated with the indicated concentrations of IFN‐γ (0 or 10 ng/mL) for 0, 6 or 48 hours. All genes were quantified by real‐time RT‐PCR experiments. The expression of STAT1(G), PRKCQ‐AS1 (H), SH3PXD2A‐AS1 (I) and CERNA2 (J) was determined in HaCaT cells with 10 ng/ml IFN‐γ stimulation for indicated time‐points. (K‐Y) The expression of 3 lncRNAs was knockdown (KD) by siRNA oligos. PRKCQ‐AS1 (K), SH3PXD2A‐AS1 (L) and CERNA2 (M) were KD with at least one siRNA oligo. Fold changes of STAT1 mRNA in HaCaT transfected with siRNAs for screening. STAT1 were significantly down‐regulated upon knockdown of PRKCQ‐AS1 (N), SH3PXD2A‐AS1 (O) and CERNA2 (P). (Q‐Y) Knockdown of 3 selected lncRNAs decrease the inflammation level in HaCaT cells with IFN‐γ treatment. The expression of CXCL9 (Q‐S), CXCL10 (T‐V) and CXCL11(W‐Y) was significantly reduced by RNAi‐mediated depletion of PRKCQ‐AS1 (Q, T, W), SH3PXD2A‐AS1 (R, U, V) and CERNA2 (W, X, Y). All experiments were performed in triplicate and the average expression level was reported. Significant differences were determined using the Mann‐Whitney unpaired two tailed *t* test. (*indicates significance, *P* < .05; **indicates significance, *P* < .01; ***indicates significance, *P* < .001)

Interferons (IFNs) play an important role in innate immune responses through IFN‐γ signalling and activation of intracellular JAK‐STAT signalling pathway.^43^ The cultured epidermal keratinocytes with IFN‐γ stimulation have been used to mimic the innate immune response in the progression of numerous skin diseases.^44^ We sought to validate the expression and functional role of lncRNA in RNA‐seq data set and external clinical samples. As a result, we found PRKCQ‐AS1, SH3PXD2A‐AS1 and CERNA2 are significantly up‐regulated in psoriasis skin compared to healthy skin (Figure 3A‐C). Expectedly, the expression of PRKCQ‐AS1 and SH3PXD2A‐AS1 was markedly elevated in psoriatic skin tissues (n = 10) compared with non‐psoriatic skin tissues (n = 10), except CERNA2 (Figure 3D‐F). We observed the up‐regulation of CERNA2 in psoriatic skin compared with normal skin but not significantly due to the heterogenous of clinical samples (Figure 3F). To validate the function of lncRNAs in IFN‐γ signalling pathway and JAK/STAT pathway activation, we sought to check the expression dynamic of lncRNAs in HaCaT stimulated by IFN‐γ (Figure 3G‐J) and IFN‐a (Figure S1). Expectedly, quantitative RT‐PCR demonstrated that the expression of STAT1 was markedly up‐regulated in HaCaT cells with 10 ng/mL IFN‐γ stimulation for indicated time‐points, suggesting that the inflammatory level of HaCaT cells is indeed elevated (Figure 3G). Furthermore, we checked the expression of lncRNA with IFN cytokines stimulated HaCaT cells. As a result, PRKCQ‐AS1 (Figure 3H) and SH3PXD2A‐AS1 (Figure 3I) were significantly up‐regulated in HaCaT cells with IFN‐γ treatment, while CERNA2 was down‐regulated (Figure 3J). However, the expression of lncRNAs was not significantly different upon IFN‐a treatment (Figure S1), which demonstrates that the expression dynamic of lncRNAs fine‐tune the activity of the IFN‐γ signaling pathway in inflammation. Further, we sought to validate the functions of 3 selected lncRNAs in HaCaT cells through RNA interference (RNAi) experiments (Figure 3K‐M). To investigate whether lncRNA participate in inflammation processes upon IFN‐γ stimulation, we examined the expression level of the downstream effector genes as the readouts, such as CXCL9, CXCL10 and CXCL11. Expectedly, the expression of STAT1 and effector genes dramatically decrease in IFN‐γ treated HaCaT upon small interfering RNAs (siRNAs) knockdown, suggesting the functionality of PRKCQ‐AS1, SH3PXD2A‐AS1 and CERNA2 in regulating IFN‐γ and JAK/STAT signalling pathway activity (Figure 3N‐P) and inflammatory level (Figure 3Q‐Y). Collectively, these results showed that lncRNAs involve in the regulation of IFN‐γ/STAT pathway activity and the inflammation level in human keratinocytes.

### 3.4 LncRNAs may regulate JAK/STAT pathway through ceRNA mechanism

**FIGURE 4:**
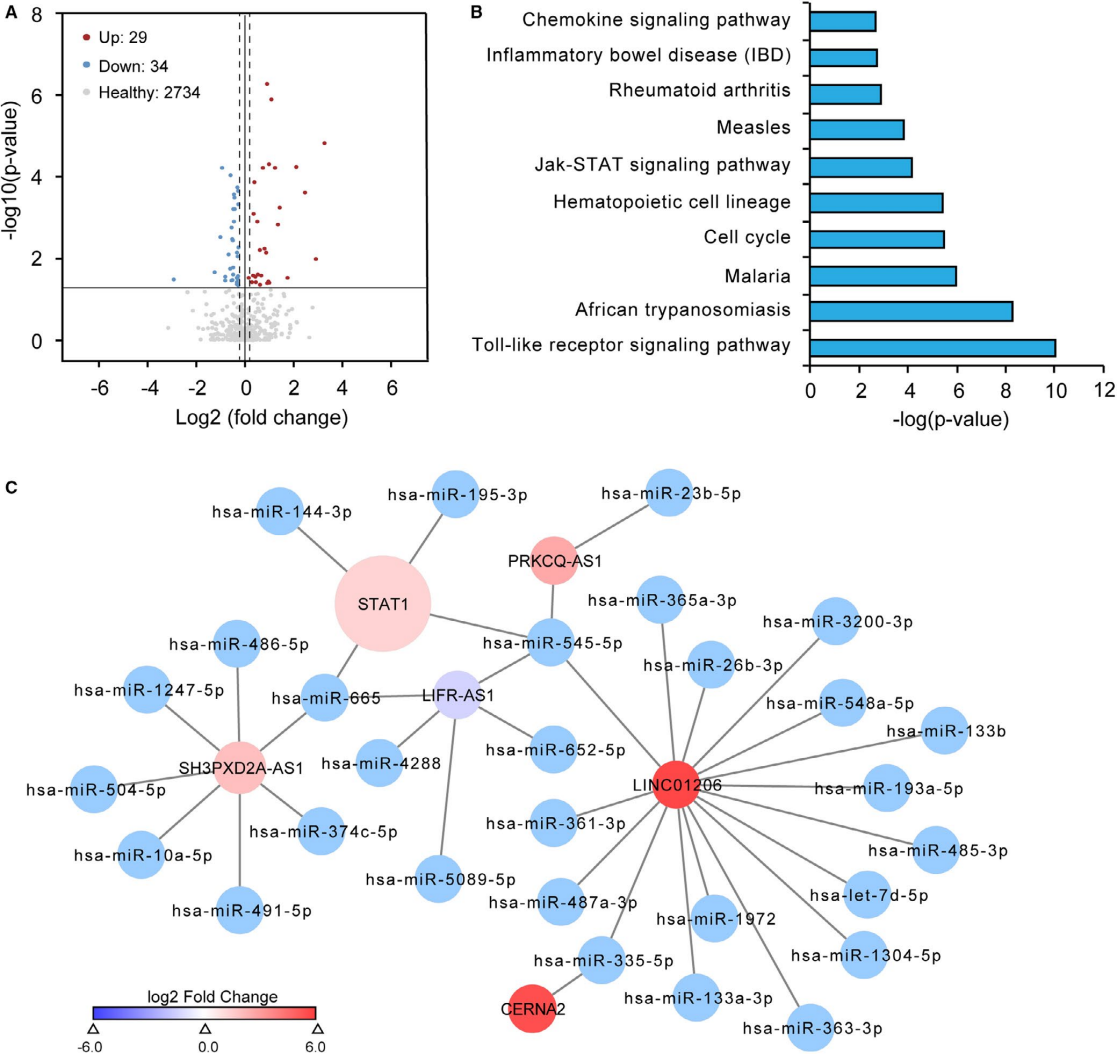
LncRNAs may regulate JAK/STAT pathway through ceRNA mechanism. A, Scatterplot showed the differentially expressed miRNAs in psoriasis compared with the healthy group. Up‐regulated miRNAs are highlighted with red, while down‐regulated miRNAs are highlighted in blue. B, KEGG pathway enrichment analysis for the target genes of differentially expressed miRNAs in psoriasis compared with the healthy group. C, The sub‐network composed of lncRNA, microRNA and mRNA involve in IFN‐γ signalling pathway JAK/STAT signalling pathway. The network consists of 35 edges among 5 lncRNA nods, 29 miRNAs and 1 mRNA

Previous studies have been demonstrated that lncRNAs act as ceRNAs to fine‐tune the expression of target genes through completing for shared miRNAs in diverse biological processes.^45^, ^46^ Thus, we hypothesized that lncRNAs might participate in the regulation of inflammation through the ceRNA mechanism. To this end, we analysed small RNA‐seq data sets from 24 psoriatic and 20 healthy skin. Interestingly, we found that 362 miRNAs were differentially expressed in psoriatic skin compared with healthy skin, including 205 up‐regulated and 157 down‐regulated miRNAs (Table S6). Then the differentially expressed microRNAs were identified with the P‐values less than 0.05 (Figure 4A). We then established putative miRNA‐lncRNAs, miRNA‐PCGs interactions using miranda, and further KEGG enrichment analysis showed that the predicted target genes of miRNAs were enriched in the JAK/STAT signalling pathway (Figure 4B; Table S7), indicating that differentially expressed lncRNAs may involve in the regulation of JAK‐STAT signalling pathway activity through ceRNA manner. To get a panorama view of ceRNA regulatory network, we constructed a ceRNA network comprising lncRNA‐miRNA‐PCG to investigate functional circuits involved in the JAK/STAT signalling pathway. We retained miRNA‐lncRNA and miRNA‐PCG pairs if they were supported with both miranda screening and miRTarbase, resulting in a credible ceRNA network consist of 392 lncRNAs, 331 miRNAs and 2131 mRNAs (Table S8). To gain more insights into the regulatory mechanism of lncRNAs, we retrieved a ceRNA sub‐network mediated by STAT1, which composited by 5 lncRNAs, 29 miRNAs and 1 PCG (Figure 4C; Table S9). Particularly, we observed potential hsa‐miR‐545‐5p target sites in PRKCQ‐AS1 and STAT1, while potential hsa‐miR‐665 target sites in SH3PXD2A‐AS1, STAT1 and LIFR‐AS1, suggesting that lncRNA may regulate the activity of JAK/STAT signalling pathway through lncRNA‐miRNA‐PCG circuitries. In summary, our analyses demonstrated that lncRNA may regulate JAK/STAT pathway through the ceRNA mechanism.

### 3.5 Experimental validation of PRKCQ‐AS1/miR‐545‐5p/STAT1 regulatory circuit

**FIGURE 5:**
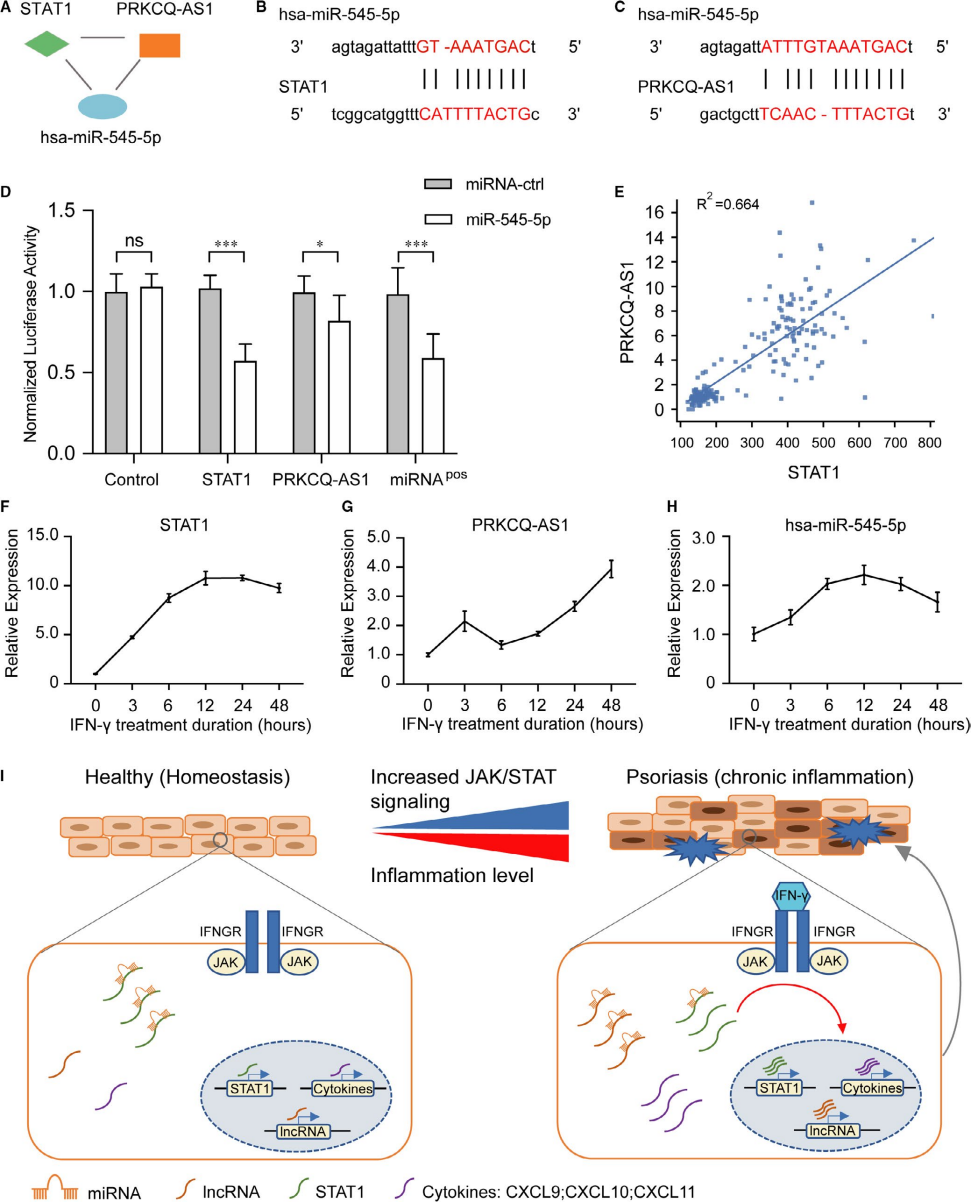
Experimental validation of PRKCQ‐AS1/miR‐545‐5p/STAT1 regulatory circuit. A, A 3‐node network motif composed of STAT1, PRKCQ‐AS1 and has‐miR‐545‐5p. B‐C, Predicted has‐miR‐545‐5p binding sites within 3’UTR of STAT1 (B) and the whole gene body of PRKCQ‐AS1 (C). D, Luciferase reporter assay for the interaction between miR‐545‐5p and PRKCQ and STAT1. The pmirGLO luciferase reporter plasmids with STAT1 mRNA coding regions and PRKCQ‐AS1 were transiently transfected into 293T cells with the miR‐545‐5p precursor and the negative control. And luciferase activities were measured after 72 hours. E, Scatter plot showed the expression of STAT1, and PRKCQ‐AS1 is positively correlated. F‐H, lncRNAs regulate the activity of JAK/STAT signalling pathway through miRNA‐mediated ceRNA mechanism. Quantitative RT‐PCR analysis showed the similar expressed pattern of STAT1 (F) and PRKCQ‐AS1 (G) upon IFN‐γ stimulation in HaCaT cells, and the expression of has‐miR‐545‐5p (H) is up‐regulated. I, A schematic mechanistic model of lncRNA functions in psoriasis. In healthy skin, the expression of lncRNAs is low and the activity of JAK/STAT signalling pathway is maintained in a low level in keratinocytes; when the skin suffers chronic inflammatory stimulation, the expression of lncRNAs is elevated and lncRNAs fine‐tuning the expression level of the key factors in JAK/STAT signalling pathway by competing for miRNAs, resulting the decreased suppression of JAK/STAT signalling pathway‐associated genes. Then, it then results the activation of JAK/STAT signalling pathway and lead to the secretion of cytokines in keratinocytes and exacerbate the inflammation level in psoriatic skin

Richard et al have demonstrated that thousands of lncRNAs are up‐regulated in psoriatic skin compared with healthy skin.^47^ However, only a small proportion of them have been experimentally tested, lots of them warranting further investigations. Therefore, we sought to validate the lncRNA‐miRNA‐JAK/STAT pathway regulatory circuit comprised of PRKCQ‐AS1, has‐miR‐545‐5p and STAT1 using HaCaT cell in vitro (Figure 5A). Miranda target site analyses showed that STAT1 and PRKCQ‐AS1 contain the binding sites of has‐miR‐545‐5p, indicating that PRKCQ‐AS1 may regulate STAT1 expression through competing for has‐miR‐545‐5p (Figure 5B‐C). Furthermore, we performed luciferases assay to validate the binding events between miR‐545‐5p and PRKCQ and STAT1 (Figure 5D). The relative luciferase activity of STAT1 mRNA and PRKCQ‐AS1 was obviously reduced after co‐transfection of miR‐545‐5p in HEK293T cells, indicating that PRKCQ‐AS1 functions as a ceRNA sponge for miR‐545‐5p to fine‐tune the gene expression of targeted genes. Further expression analysis again confirmed a strong correlation between STAT1 and PRKCQ‐AS1 (Figure 5E). To test this, we checked the expression dynamic of PRKCQ‐AS1, STAT1 and has‐miR‐545‐5p during the differentiation process of HaCaT upon IFN‐γ stimulation. As a result, we observed that the expression of STAT1 and PRKCQ‐AS1 was activated in response to IFN‐γ treatment but has‐miR‐545‐5p remained constant (Figure 5F‐H), suggesting that PRKCQ‐AS1 and STAT1 are strongly associated with the activity of JAK/STAT signalling pathway and has‐miR‐545‐5p act as a brake to modulate this process. Interestingly, the expression of PRKCQ‐AS1 dramatically increased in 12 hour after IFN‐γ treatment, while STAT1 was activated as early as 6h (Figure 5F‐H). It means that has‐miR‐545‐5p act as a feedback regulator to fine‐tune the expression of STAT1, while PRKCQ‐AS1 act as a sponge to control the abundance of miRNAs, suggesting PRKCQ‐AS1 acts as competing endogenous RNA for regulating the activity of JAK/STAT signalling pathway. Taken together, our experimental analysis showed that dysregulation of the ceRNA circuit comprised of PRKCQ‐AS1, has‐miR‐545‐5p and STAT1 act as a vital regulatory role in JAK/STAT signalling pathway activation, which involve in the hyper‐inflammation of keratinocytes in psoriasis (Figure 5I).

## 4 DISCUSSION

Psoriasis is a chronic autoimmune disease with complex genetic architecture. Previous genome‐wide association studies (GWAS) have showed that numerous susceptibility loci and a large majority of susceptibility loci are at non‐coding region of the human genome, indicating the important role for non‐coding region in the progression of psoriasis.^47^, ^48^ Shat et al showed that extensive lncRNA are differentially expressed in psoriatic skin compared with healthy skin.^49^ However, the underline mechanisms of lncRNA in the progression of psoriatic require further investigations.

Here, we found that 156 down‐regulated and 69 up‐regulated lncRNAs in psoriasis skin compared with healthy skin. Then, lncRNA‐PCGs co‐expression analysis demonstrated that PCGs associated up‐regulated lncRNAs of psoriasis were enriched in IFN‐γ signalling pathway. Further experimental validations showed that lncRNAs indeed involved in the regulation of IFN‐γ signalling pathway. To gain more insights into lncRNA regulatory mechanism, microRNA target screening analysis demonstrated that lncRNAs and JAK/STAT pathway‐associated genes co‐regulated by the same set of miRNAs, suggesting lncRNAs may regulate JAK/STAT pathway through competing for the shared miRNAs. Furthermore, we experimentally validate lncRNA‐miRNA‐JAK/STAT pathway regulatory circuits in HaCaT cells. Thus, our computational analyses and experimental validations showed that lncRNA plays an important role in the onset of psoriasis through mediated JAK‐STAT signalling pathway.

LncRNA serve important roles in regulating various functions in the immune system.^45^ Our result confirmed that lncRNA dysregulation is associated with a variety of cell signalling pathways and plays a vital role in the pathogenesis of psoriasis. Previous studies have showed that signalling pathways regulated by lncRNA may modulate multiple biological processes.^28^ JAK/STAT signalling was a critical pathway for progression of inflammatory and autoimmune diseases, such as rheumatoid arthritis, psoriasis and inflammatory bowel disease.^11^, ^12^ In the present study, the co‐expression results support a network model that lncRNA and protein together to trigger dysregulation of JAK/STAT signalling pathway in psoriasis. Those findings suggested that lncRNA potential acts its function through interacting with RNA binding proteins. We then investigated the regulatory associations among lncRNA and the JAK/STAT pathway by integrative analysis of the lncRNA‐miRNA‐PCG network. Interestingly, we found that the differentially expressed lncRNA, miRNAs and PCGs form 3‐node network motifs to modulate the JAK/STAT pathway activity through ceRNA mechanism. Notably, most of the differentially expressed lncRNA and PCG pairs are regulated by the same set of miRNAs in psoriasis, indicating that lncRNAs regulate the targeted PCGs abundance through completing interaction with miRNAs. The established ceRNA network and network motif analysis methods will benefit further mechanistic investigations on psoriasis and other inflammatory disorders.

Current study determined the function of lncRNAs in the regulation of JAK/STAT pathway in psoriasis. To validate the network analysis results and investigate the potential role of lncRNA in psoriasis, 3 dysregulated lncRNA associated with JAK/STAT pathway were randomly selected for RT‐qPCR analysis. Furthermore, we found that the expression of PRKCQ‐AS1 and SH3PXD2A‐AS1 is elevated in HaCaT cells stimulated by interferon gamma (IFN‐γ). Those results conferred that lncRNAs are activated in inflammatory skin, while the increased expression of lncRNA modulates the activities of JAK/STAT pathways. The up‐regulation of lncRNAs amplify the expression of key regulators in JAK/STAT signalling pathway and activate autoimmune responses, eventually lead to hyper‐inflammation in psoriatic skin (Figure 5I). In summary, the present study enhances our understanding of lncRNA function in psoriasis. Moreover, we validated that dysregulation of lncRNA‐JAK/STAT pathway axis plays an important role in the pathogenesis of psoriasis and thus provide potential therapeutic targets for psoriasis treatments.

## CONFLICT OF INTEREST

The authors confirm that there are no conflicts of interest.

## AUTHOR CONTRIBUTIONS

Conceptualization, Yongfeng Chen; Formal analysis, Jingxia Lin; Data curation, Xuefei Li; Funding acquisition, Yongfeng Chen; Investigation, Jingxia Lin and Xuefei Li; Methodology, Xuefei Li; Validation, Jingxia Lin, Fangfei Zhang and Lei Zhu; Writing – original draft, Jingxia Lin; Writing – review & editing, Yongfeng Chen.

## Supporting information

Fig S1

Table S1

Table S2

Table S3

Table S4

Table S5

Table S6

Table S7

Table S8

Table S9
