# Supplementary figures and images for: Transcriptome wide analysis of long non‐coding RNA‐associated ceRNA regulatory circuits in psoriasis
Source: J Cell Mol Med. 2021 Jun 2;25(14):6925–35. doi: 10.1111/jcmm.16703 (PMC8278092; doi:10.1111/jcmm.16703)

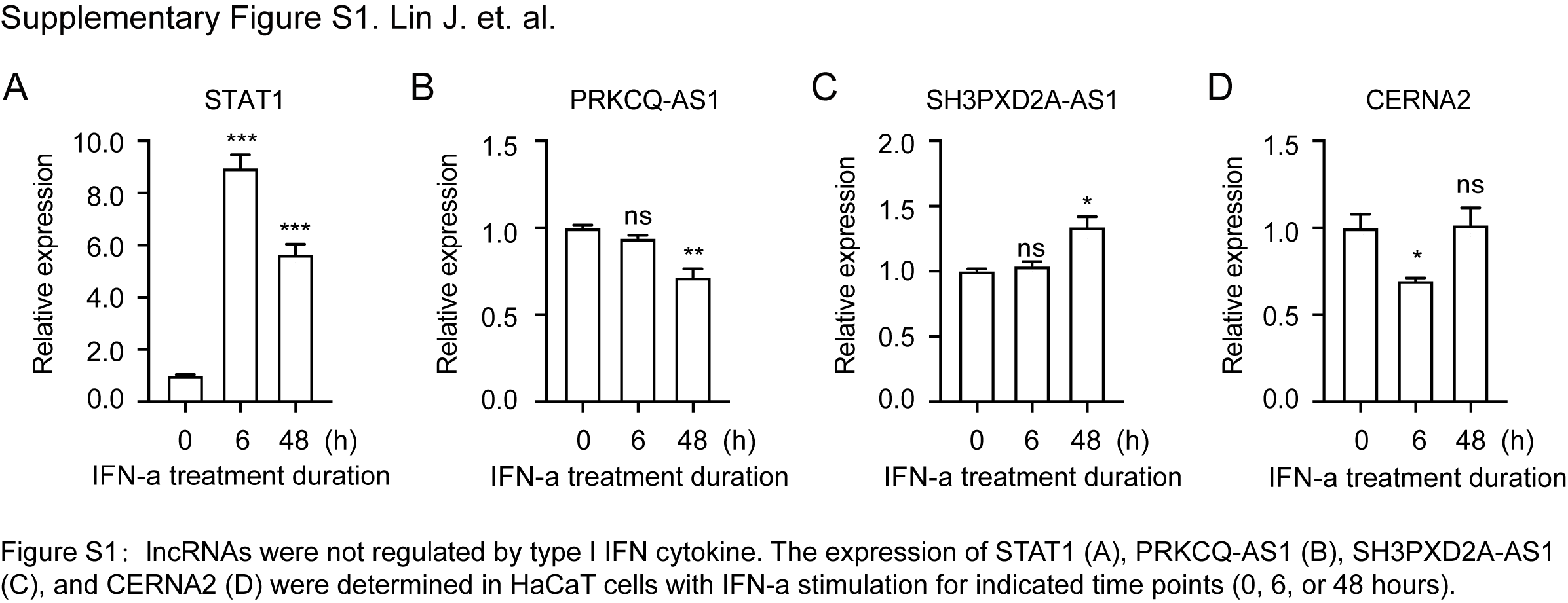

Supplement: Supplementary file 1 — Fig S1 [file JCMM-25-6925-s002.tif]
